# MicroRNA‐132 is overexpressed in fetuses with late‐onset fetal growth restriction

**DOI:** 10.1002/hsr2.558

**Published:** 2022-03-15

**Authors:** José Morales‐Roselló, Gabriela Loscalzo, Eva María García‐Lopez, José Luis García‐Gimenez, Alfredo Perales‐Marín

**Affiliations:** ^1^ Servicio de Obstetricia y Ginecología Hospital Universitario y Politécnico La Fe Valencia Spain; ^2^ Departamento de Pediatría, Obstetricia y Ginecología Universidad de Valencia Valencia Spain; ^3^ EpiDisease SL, and Consortium Center for Biomedical Network Research on Rare Diseases (CIBERER) Institute of Health Carlos III Valencia Spain; ^4^ Departamento de Fisiología Universidad de Valencia Valencia Spain

**Keywords:** Doppler ultrasound, late‐onset fetal growth restriction, microRNA, miR‐132

## Abstract

**Background and Aims:**

To evaluate the expression of microRNA 132 (miR‐132) in fetuses with normal growth and in fetuses with late‐onset growth restriction (FGR).

**Methods:**

In a prospective cohort study, 48 fetuses (24 with late‐onset FGR and 24 with normal growth) were scanned with Doppler ultrasound after 34 weeks to measure the umbilical artery and middle cerebral artery pulsatility indices and followed until birth. Subsequently, blood samples from the umbilical cord were collected to evaluate the expression of miR‐132 by means of Real‐time quantitative polymerase chain reaction, determining the existence of normality cut‐offs and associations with birth weight (BW) centile, cerebroplacental ratio multiples of the median (CPR MoM), and intrapartum fetal compromise (IFC).

**Results:**

In comparison with normal fetuses, late‐onset FGR fetuses showed upregulation of miR‐132 (33.94 ± 45.04 vs. 2.88 ± 9.32 2−dd*C*_t_, *p* < 0.001). Using 5 as a cut‐off we obtained a sensitivity of 50% and a specificity of 96% for the diagnosis of FGR, while for IFC these values were respectively 27% and 73%. Expression of miR‐132 was associated with BW centile but not with CPR MoM. Finally, the best detection of IFC was achieved combining miR‐132 expression and CPR MoM (AUC = 0.69, *p* < 0.05).

**Conclusion:**

Fetuses with late‐onset FGR show upregulation of miR‐132. Further studies are needed to investigate the role of miR‐132 in the management of late‐onset FGR.

## 1 INTRODUCTION

Late‐onset fetal growth restriction (FGR) is characterized by an unbalance between fetal demands and placental supply occurring after Week 32, which results in low cerebroplacental ratio (CPR) values regardless of fetal birth weight (BW).^1^, ^2^ Fetal hypoxia in late‐onset FGR is usually mild and subtle, with hemodynamic changes limited to the middle cerebral and umbilical arteries.^1^ However, it can also cause perinatal mortality and morbidity.^3^ And just as importantly, it can lead to suboptimal dendritic formation, and poorer postnatal cognitive status.^4^, ^5^ Consequently, detection of late‐onset FGR and intrapartum fetal compromise (IFC) has become of key importance to prevent neurological long‐term consequences. Unfortunately, current models based on estimated fetal weight and Doppler ultrasound (umbilical, middle cerebral, and uterine arteries pulsatility indices), alone or combined with serological markers such as placental growth factor, have not proven to be accurate enough for clinical diagnosis,^6^, ^7^, ^8^, ^9^ making the search for new determinants of FGR and IFC a key issue in fetal medicine.

MicroRNAs (miRNAs) are small RNA sequences with the ability to regulate gene expression by means of inhibition of translation or promotion of messenger RNA (mRNA) degradation.^10^, ^11^, ^12^, ^13^ Cell culture and clinical experimentation have evidenced several miRNAs related to neuronal function.^14^, ^15^ Among them outstands miRNA‐132 (miR‐132) for its important role in maintaining and promoting neuronal activity: In fact, miR‐132 behaves physiologically as a dynamic regulator of cognitive capacity, which is required not only for dendrite and spine maturation but also for synaptic regulation and function.^16^, ^17^, ^18^, ^19^, ^20^, ^21^ Moreover, in patients with Alzheimer's, miR‐132 protects neurons against amyloid‐β (Aβ) and glutamate excitotoxicity and mitigates tau pathology.^22^, ^23^ A protection was also observed in patients with Parkinson's^24^ and Huntington's disease,^25^ where it has been evaluated as a treatment for relieving symptoms and delaying disease progression.

Considering this background and to investigate new markers of chronic fetal hypoxia and brain damage, we aimed to evaluate miR‐132 expression in fetuses with late‐onset FGR, studying potential roles in the diagnosis of FGR and IFC.

## 2 METHODS

### 2.1 Study design

This was a prospective cohort study of 48 fetuses, belonging to the area controlled by the maternity of the public tertiary hospital La Fe (Institution Review Board and Hospital Ethics Committee permission number 2016/0453). To avoid overlapping with early‐onset FGR cases, these fetuses underwent an ultrasound scan, between 34 and 41 weeks, which included a Doppler interrogation of the umbilical artery pulsatility index (UA PI), middle cerebral artery pulsatility index (MCA PI), and CPR (a ratio which reflects the relationship between fetal demands and placental supply). UA PI and MCA PI were recorded using color and pulse Doppler according to earlier descriptions,^26^, ^27^ while CPR was calculated as the simple ratio between MCA PI and UA PI.^28^ All pregnancies were followed‐up and delivered in 15 days or less after the scan, between 35 and 41 weeks, and only the last examination per fetus was included. To adjust for the effect of the gestational age (GA), BW values were converted into local reference centiles^29^ adjusting also for fetal gender, and CPR values were converted into multiples of the median (MoM) dividing each Doppler value by the 50th centile at each gestational age as earlier described.^26^ CPR medians (50th centile) were represented by the equation:

$$\text{CPR 50th centile}\;=-3.814786276+0.36363249\times \text{GA}-0.005646672\times {\text{GA}}^{2}$$

Where GA was gestational age in weeks with decimals.

All Doppler examinations were performed by the first author, a certified teaching expert in obstetric ultrasound by the Spanish Society of Obstetrics and Gynecology, using General Electric Voluson® (E8/E6/730) ultrasound machines (General Electric Healthcare) with 2–8 MHz convex probes, during fetal quiescence, in the absence of fetal tachycardia, and keeping the insonation angle with the examined vessels as small as possible and always below 30°. GA was determined according to the crown‐rump length in the first trimester. Multiple pregnancies and those complicated by congenital fetal abnormalities were excluded. Gestational characteristics including maternal age, weight, height, body mass index (BMI), parity, number of gestations, and ethnicity were collected at examination together with ultrasound parameters, while labor data including BW, BW centile, mode of delivery, 5 min Apgar score, cord arterial pH and baby destiny were collected at birth.

### 2.2 Study population

For comparison purposes, the study evaluated two groups of fetuses:

1. Late‐onset FGR: characterized by a BW <3rd centile or alternatively a BW between the 3rd and 10th centile plus an abnormal fetal Doppler (represented by a CPR < 0.6765 MoM).^29^, ^30^, ^31^, ^32^, ^33^
2. Normal fetuses: characterized by a BW >3rd centile plus a normal Doppler (represented by a CPR > 0.6765 MoM). For study purposes, small for gestational age fetuses (BW between the 3rd and 10th centile plus a normal fetal Doppler) were considered within normality limits.

Other fetuses, like those with normal BW plus an abnormal CPR, were not included in the study, although they represented an interesting group for future research.^30^, ^31^, ^32^, ^33^, ^34^

IFC was considered when any of the following circumstances were present: (1) abnormal intrapartum fetal heart rate (according to the intrapartum fetal monitoring guidelines of the FIGO),^35^ (2) intrapartum fetal scalp pH <7.20 requiring cesarean section, and (3) neonatal umbilical cord pH <7.20.

To avoid biases, we did not consider 5 min Apgar score and postpartum admission to neonatal care for outcome analysis due to their close relationship with BW centile. Finally, the onset of labor occurred for obstetric indications, and management was done as per local protocol according to fetal progression at labor.

### 2.3 Real‐time quantitative polymerase chain reaction (qPCR) from umbilical cord plasma

Blood samples were collected from all fetuses in ethylenediaminetetraacetic acid tubes just after delivery. Each one was centrifuged at 2000–2500 rpm for 10 min to separate the plasma, and this was stored at −80°C until RNA extraction. Cell‐free total RNA (including miRNAs) was isolated from 500 µl of plasma using the miRNeasy Serum/Plasma kit (Qiagen®), following the manufacturer's protocol. The concentration of cell‐free total RNA (including miRNAs) was subsequently quantified using NanoDrop One® UV‐spectrophotometer (Thermo Scientific).

Reverse transcription reactions were performed using the TaqMan® miRNA Reverse Transcription Kit (Part No. 4366596; Applied Biosystems Inc.) and miRNA‐specific stem‐loop primers (Part No. 4366597; Applied Biosystems Inc.) and 100 ng of input cell‐free RNA in a 20 µl RT reaction. Real‐time PCR reactions were performed in triplicate, in scaled‐down 10 µl reaction volumes using 5 µl TaqMan® 2× Universal PCR Master Mix (Applied Biosystems Inc.) with No UNG, 0.5 µl TaqMan® Small RNA assay (20×) (Applied Biosystems Inc.), (hsa‐miR‐132 [Assay ID 000457]), 3.5 µl of nuclease‐free water, and 1 µl of RT product. Real‐time PCR was carried out on an Applied BioSystems QuantStudio5® thermocycler (Applied Biosystems Inc.). We used hsa‐miR‐191‐5p (Assay ID 002299), which has been previously used as an endogenous control to normalize the expression of miRNAs in plasma samples. All the fold‐change data were obtained using the delta‐delta *C*_t_ method ($2^{‐∆∆C_{t}}$ or 2−dd*C*_t_).^36^

### 2.4 Statistical analysis

Continuous and categorical variables in the study populations, including the expression of miR‐132, were compared using Mann–Whitney *U* and Fisher tests. Afterward, values were depicted in scattergrams and the presence of associations with BW centile or CPR MoM was evaluated, including the existence of cut‐offs differentiating normal from abnormal fetuses. Finally, the different accuracies for the diagnosis of IFC were studied by means of ROC analysis, determining the areas under the curve (AUC) and the 95% confidence intervals (95% CI). *p* < 0.05 were considered significant. Graphs and statistics were performed using GraphPad Prism® 5.0a and StatPlus® Pro 7.3.3.2 for Apple Macintosh.

## 3 RESULTS

Concerning the characteristics of the study population, in summary, it included 48 fetuses scanned after 34 weeks, of which 19 (39.6%) were males. The mean maternal age, gravidity, parity, and BMI were 32.5, 2, 0.5, and 23, and the mean GA at examination and delivery was 38.5 and 39.1 weeks. Most pregnancies initiated labor with induction (79.2%), delivering spontaneously (47.9%), and most neonates were born uneventfully, accompanying the mother to the maternity ward (85.4%). Moreover, despite the important proportion of fetuses with growth restriction (58%), no fetus presented severe hypoxia, suggested by an Apgar score below 7 at 5 min or cord pH below 7.10.

Table 1 compares normal versus FGR pregnancies. In summary, fetuses in the FGR group were examined and delivered earlier (*p* < 0.01), had a lower CPR MoM, BW, and BW centile (*p* < 0.0001) and presented worse perinatal outcome (*p* < 0.01) which required more frequent postnatal pediatric surveillance.

**Table 1:** Comparisons between normal and late‐onset fetal growth restriction (FGR) populations

Variable	Late‐onset FGR (*N* = 24) (50%)	Normal fetuses (*N* = 24) (50%)	
Continuous/Categorical data	Mean (SD), median (1st, 3rd Quartiles)/*N* (%)	Mean (SD), median (1st, 3rd Quartiles)/*N* (%)	*p* value*/**
Maternal characteristics			
Maternal age (years)	31.5 (5.2), 31.0 (29, 34.5)	34.0 (4.2), 34 (31.0, 36)	NS
Gravidity	1.7 (1.0), 1 (1, 2)	2.2 (1.4), 2 (1, 3)	NS
Parity	0.4 (0.6), 0 (0, 1)	0.5 (0.6), 0 (0, 1)	NS
Maternal prepregnancy weight (kg)	58.8 (9.2), 58.5 (50.5, 66.7)	61.5 (10), 62 (53.5, 69.2)	NS
Maternal height (cm)	162.6 (5.6), 161.5 (158.3, 167.8)	160.9 (8.3), 161.5 (154.5, 167)	NS
Maternal body mass index	22.2 (3), 22.5 (20.1, 24.7)	23.7 (3.4), 23.7 (20.8, 26.6)	NS
Smoking during pregnancy	8 (33.3)	4 (16.7)	NS
Fetal examination			
Fetal gender (male)	9 (37.5)	10 (41.7)	NS
Gestational age at ultrasound examination (week)	37.9 (1.5), 37.3 (36.7, 38.7)	39.1 (1), 39.1 (38.6, 40)	<0.01
Gestational age at delivery (week)	38.4 (1.5), 37.9 (36.4, 39.9)	39.7 (1.2), 39.7 (38.8, 40.8)	<0.01
Interval examination‐delivery (day)	3.5 (2.8), 3 (1.2, 5)	4.3 (3.5), 4 (1.2, 6)	NS
CPR MoM	0.70 (0.26), 0.62 (0.49, 0.96)	1.2 (0.3), 1.2 (1, 1.4)	<0.0001
Birth weight (g)	2278 (320), 2340 (2053, 2538)	3179 (453), 3145 (2753, 3575)	<0.0001
Birth weight centile	1.5 (1.9), 1 (0, 2)	35.3 (26.4), 29.5 (13.75, 57.5)	<0.0001
Gestational age at ultrasound examination (week)	37.9 (1.5), 37.3 (36.7, 38.7)	39.1 (1), 39.1 (38.6, 40)	<0.01
Gestational age at delivery (week)	38.4 (1.5), 37.9 (36.4, 39.9)	39.7 (1.2), 39.7 (38.8, 40.8)	<0.01
Labor and delivery			
IFC	11 (45.8)	2 (8.3)	<0.01
Apgar <7 at 5 min	0 (0)	0 (0)	NS
Arterial pH <7.20	6 (25)	3 (12.5)	NS
Arterial pH <7.10	0 (0)	0 (0)	NS
Type of event leading to labor onset			
Spontaneous labor onset	2 (8.3)	6 (5)	NS
Induction of labor	21 (87.5)	17 (70.8)	NS
Elective cesarean	1 (4.2)	1 (4.2)	NS
Via of delivery			
Cesarean section (scheduled)	1 (4.2)	1 (4.2)	NS
Cesarean section (failure to progress)	2 (8.3)	2 (8.3)	NS
Cesarean section (abnormal CTG)	7 (29.2)	2 (8.3)	NS
Assisted vaginal delivery	6 (25)	4 (16.7)	NS
Spontaneous vaginal delivery	8 (33.3)	15 (62.5)	NS
Neonate destiny			
Maternal ward	17 (70.8)	24 (100)	<0.01
Neonates ward	7 (29.2)	0 (0)	<0.01
Pediatric Intensive care unit	0 (0)	0 (0)	NS

Abbreviations: BW, birth weight; CPR MoM, cerebroplacental ratio multiples of the median; FGR: fetal growth restriction: BW <3rd centile or alternatively BW <10th centile plus abnormal Doppler (represented by a CPR < 0.6765 MoM), IFC: intrapartum fetal compromise; IFC, intrapartum fetal compromise; MoM, multiples of the median, *Mann–Whitney *U* test, **Fisher test; SD, standard deviation.

**Figure 1:**
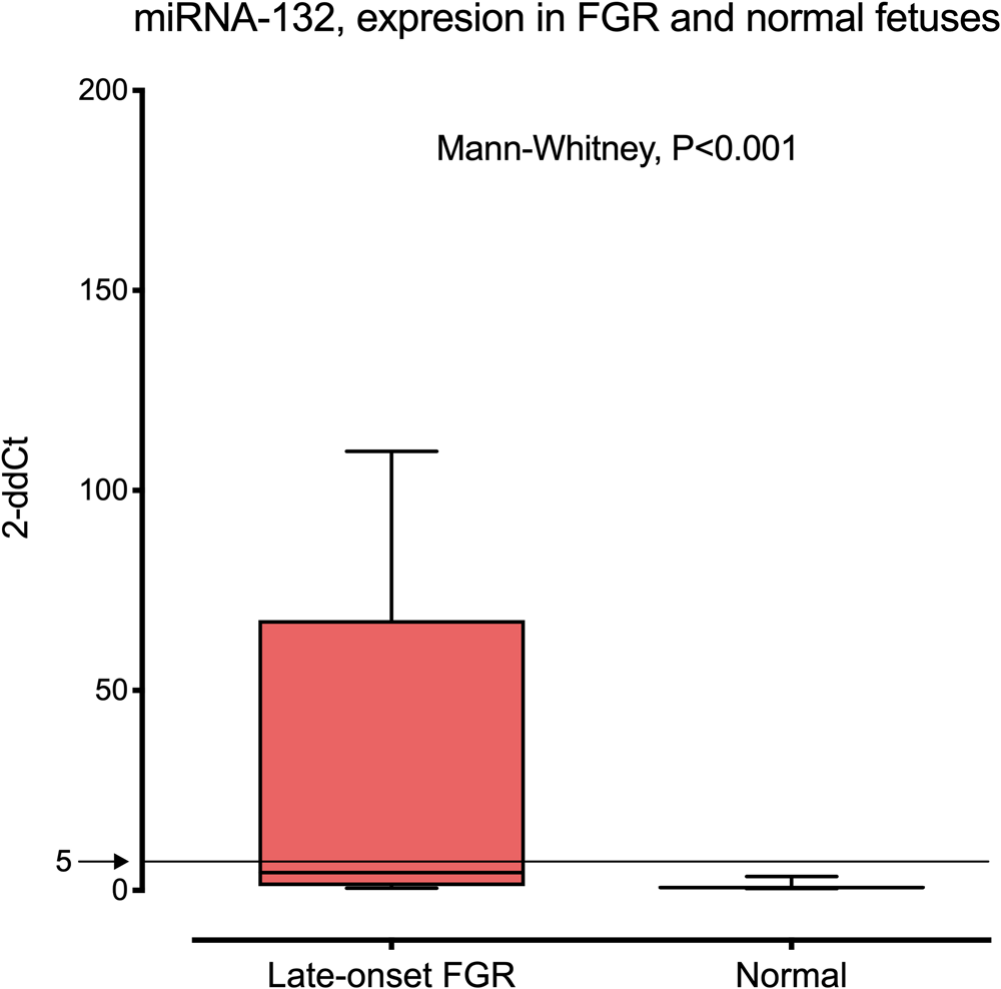
Expression of miR‐132 in normal fetuses and in fetuses affected with late‐onset growth restriction (FGR), (in red). Fetuses in the FGR group presented a significantly higher miR‐132 expression (*p* < 0.001). Boxes represent the median and interquartile range. Whiskers represent the 90th and 10th centiles. Most fetuses in the normal group presented miR‐132 expressions below 5 2−dd*C*_t_

Figure 1 shows the expression of miR‐132 in FGR and normal fetuses. Fetuses in the late‐onset FGR group presented a significantly higher miR‐132 expression (*p* < 0.001). Moreover, most fetuses in the normal group presented miR‐132 expressions ($2^{‐\mathrm{dd}C_{t}}$) below 5.

**Figure 2:**
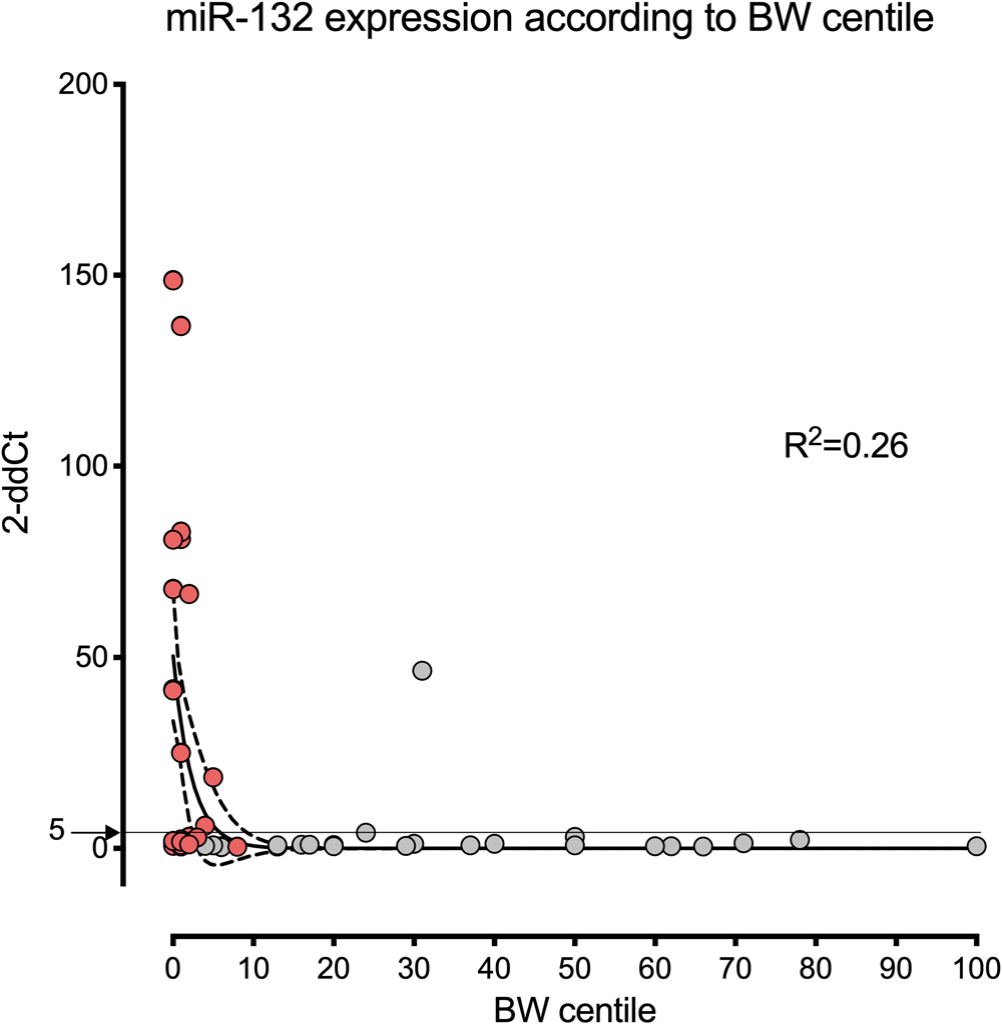
Scattered values of the study population depicted according to birth weight (BW) centile and miR‐132 expression (2−dd*C*_t_). A correlation was detected between miR‐132 expression and BW centile (*R* ^2^ = 0.26). Lines represent the exponential correlation with its 95% confidence interval. Most of the cases with miR‐132 overexpression present low BW centiles. Most fetuses in the normal group presented miR‐132 expressions below 5 2−dd*C*_t_

Figure 2 shows the scattered values of the study population depicted according to BW centile and miR‐132 expression. Most of the miR‐132 cases with overexpression are correlated with lower BW centiles, existing a clear correlation between both parameters (*R* ^2^ = 0.26). Moreover, the use of 5 (2−dd*C*_t_) as the cut‐off value for miR‐132 expression showed a sensitivity of 50% and a specificity of 96%, for the diagnosis of FGR and a sensitivity of 27% and a specificity of 73% for the diagnosis of IFC.

**Figure 3:**
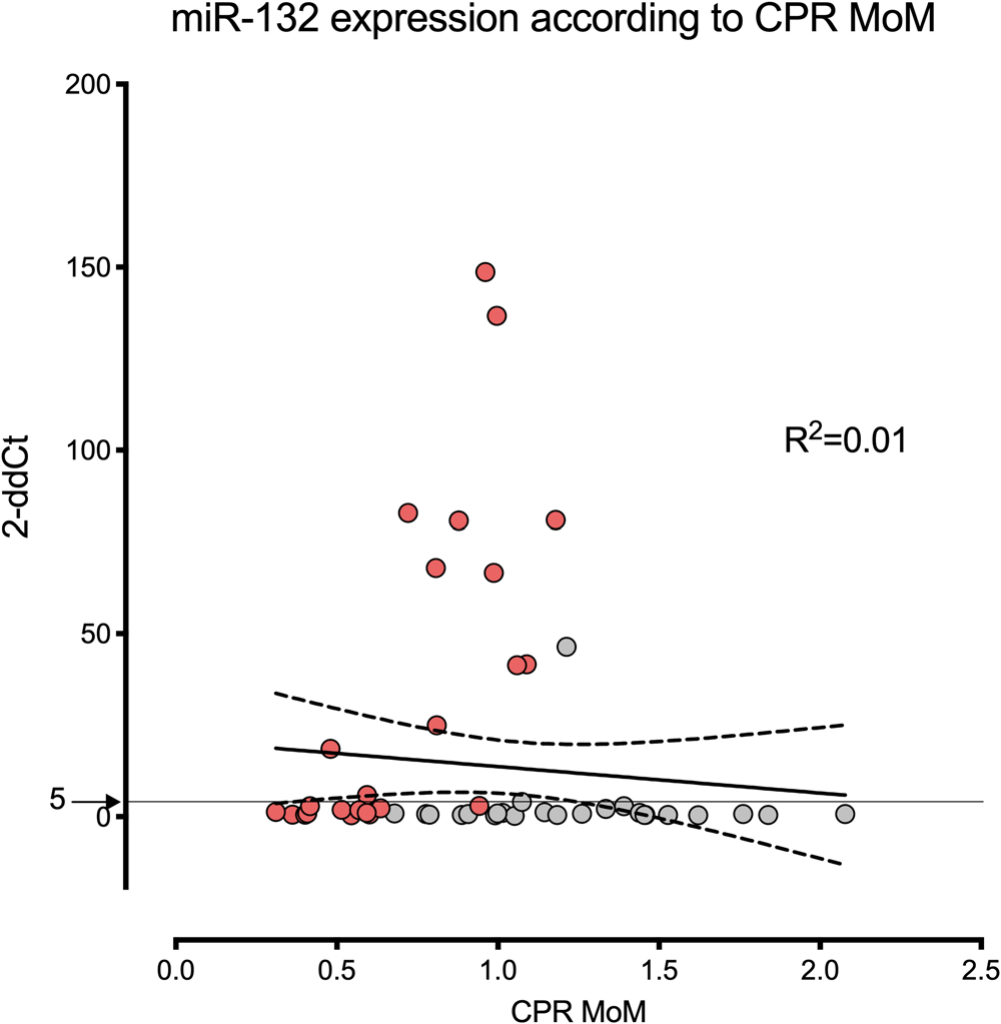
Scattered values of the study population depicted according to cerebroplacental ratio multiples of the median (CPR MoM) and miR‐132 expression (2−ddC_t_). No correlation was detected between miR‐132 expression and CPR MoM (*R* ^2^ = 0.01). Lines represent the linear correlation with its 95% confidence interval. Most fetuses in the normal group presented miR‐132 expressions below 5 2−dd*C*_t_

Figure 3 shows the scattered values of the study population depicted according to CPR MoM and miR‐132 expression (2−dd*C*_t_). No correlation was detected between miR‐132 expression and brain sparing represented by CPR MoM (*R* ^2^ = 0).

Table 2 shows the accuracies of several models (single and combined) for the diagnosis of IFC. Models that included only single parameters presented poor AUCs and were not significant, probably due to the low number of cases. Concerning combined models, only those including CPR MoM were significant although with moderate AUC (0.69, *p* < 0.05). The addition of miR‐132 expression to CPR MoM seemed to improve IFC detection (AUC from 0.65 up to 0.69), while the effect of BW centile addition seemed to be null, suggesting miR‐132 expression plus CPR MoM was the optimal combination for the detection of IFC.

**Table 2:** Accuracy of several models for the prediction of intrapartum fetal compromise (IFC)

Logistic regression model	AUC	Lower 95% CI	Upper 95% CI	*p* Value
miR‐132 alone	0.60	0.40	0.80	NS
CPR MoM alone	0.65	0.47	0.82	NS
BW centile alone	0.64	0.46	0.81	NS
CPR MoM + miR‐132	0.69	0.51	0.86	**P** < **0.05**
CPR MoM + BW centile	0.65	0.47	0.82	NS
miR‐132 + BW centile	0.62	0.45	0.80	NS
CPR MoM + BW centile + miR‐132	0.69	0.58	0.87	**P** < **0.05**

*Note*: The best model included cerebroplacental ratio multiples of the median (CPR MoM) and expression of miR‐132 in neonatal cord blood. No benefit was obtained adding birth weight (BW) centile.

Abbreviations: AUC, areas under the curve; CI, confidence interval.

## 4 DISCUSSION

### 4.1 Principal finding

Analysis of umbilical blood at birth by means of qPCR showed that miR‐132 was overexpressed in fetuses with late‐onset FGR. This overexpression correlated with BW centile and could be incorporated into a multivariable model to improve CPR detection of IFC.

### 4.2 Research implications

A number of miRNAs have been related to the nervous system.^37^, ^38^ However miR‐132, a member of the miR‐212/132 cluster, stands out for its importance in neuronal survival.^39^, ^40^, ^41^, ^42^, ^43^ Production of miR‐132 is crucial for neuronal function and is increased whenever the neuronal tissue is threatened, not only by fetal hypoxia^44^, ^45^ but also by other harmful agents like Bupivacain^46^, ^47^ or valproate.^48^ In this regard, despite the mechanisms for miR‐132 stimulation having not been fully described, it seems to be a common end‐point for different protection pathways, like those acting by means of acetylcholine,^49^ melatonin,^50^ and especially brain‐derived neurotrophic factors (BDNF).^51^, ^52^, ^53^, ^54^, ^55^

In view of the previous evidence, we expected in our population not only the observed association with BW centile but also some degree of correlation between miR‐132 expression and CPR MoM. However, we were surprised to see that miR‐132 expression was only correlated with BW centile.

A possible explanation for this apparent incongruence might be the existence of different sources for miR‐132 expression, not necessarily the brain in direct relation with cerebral vasodilation. Accumulating evidence indicates that miRNAs are secreted in exosomes or microvesicle‐encapsulated forms^56^, ^57^ or released in vesicle‐free forms bound to proteins.^58^ Moreover, miR‐132 production has also been related to hepatic,^59^ cardiac,^60^, ^61^ and adipose tissue^62^ activity, which are also plausible sources for miR‐132 production in fetuses with FGR. Therefore, blood levels of miR‐132 do not have necessarily reflect miR‐132 activity in the brain, which may be indeed a target for external production. In this regard and despite the well‐known effect of miR‐132 on neuronal tissue, maternal serum levels could be the resultant of miR‐132 production at different sites and might not necessarily correlate with CPR MoM.

### 4.3 Clinical implications

Regardless of its origin, we have preliminarily proved that miR‐132 overexpression occurs in FGR fetuses and that this information might be added to ultrasound to improve the prediction of IFC. However, this is of little use considering that before labor there is no access to fetal cord blood without performing invasive procedures. In this regard, it is yet to be established that this overexpression can be detected in maternal blood. However, some findings support this hypothesis: on one hand, miR‐132 is transferred via exosomes to proximal endothelial cells to maintain brain vascular integrity.^63^ On the other, hypoxia‐related miRNA produced in the placenta can cross the placental barrier and be detected in maternal blood.^64^ Therefore, if other miRNAs can circulate between the mother and the fetus, miR‐132 might also be detected in maternal serum and become a marker of outcome in an isolated or combined way. Therefore, a practical consequence of miR‐132 overexpression might be the possibility to detect differential levels in maternal blood, increasing the predictive ability for IFC. This will be the object of future research.

### 4.4 Comparison with earlier references

Regarding previous references, while we studied fetal blood in late‐onset FGR cases without pre‐eclampsia, earlier works evaluated maternal blood in early‐onset pregnancies frequently affected with pre‐eclampsia. Moreover, they did not study miR‐132.^65^, ^66^, ^67^, ^68^, ^69^, ^70^ Consequently, we could not find previous references with which to compare our work.

### 4.5 Strengths and limitations

The main strength of this study is its novelty, as we have been the first investigators to evaluate miR‐132 in fetal cord blood and the first to describe an overexpression in late‐onset‐FGR. Shortcomings are the low number of cases, which limits the ability to obtain robust conclusions, the nonspecificity of fetal blood origin as above indicated, the possibility that miRNA expression varies with GA, and the absence of postnatal follow‐up in relation to neurocognitive evolution.

In conclusion, in comparison to fetuses with normal growth, fetuses with late‐onset FGR present a fold difference which suggests upregulation of miR‐132 in cord blood serum. Future studies are needed to investigate the role of miR‐132 expression in the diagnosis and management of late‐onset FGR.

## CONFLICTS OF INTEREST

The authors declare no conflicts of interest. The funding source (MarieSkłodowska‐Curie grant agreement number 765274) had not any involvement in any conflicts of interest.

## AUTHOR CONTRIBUTIONS

José Morales‐Roselló (https://orcid.org/0000-0002-8783-6710) designed the study, performed the ultrasound examinations, performed part of the statistical analysis, and wrote the manuscript. Eva María García Lopez and José Luis García Gimenez made the qPCR analysis, supervised the final manuscript, and suggested valuable inputs to the text. Gabriela Loscalzo performed data search and made notable contributions to the final text. Alfredo Perales Marín supervised the manuscript and suggested valuable inputs to the text. All authors have read and approved the final version of the manuscript. The corresponding author had full access to all the data in this study and takes complete responsibility for the integrity of the data and the accuracy of the data analysis.

## Data Availability

Data are available from the corresponding author upon request. The authors report no conflict of interest.
